# *PARK2* inhibits osteosarcoma cell growth through the JAK2/STAT3/VEGF signaling pathway

**DOI:** 10.1038/s41419-018-0401-8

**Published:** 2018-03-07

**Authors:** Zhong Lei, Huijie Duan, Tengfei Zhao, Yuxiang Zhang, Guoqi Li, Jiahong Meng, Suzhan Zhang, Weiqi Yan

**Affiliations:** 1 Department of orthopedics Research Institute, The Second Affiliated Hospital, School of Medicine, Zhejiang University, Hangzhou, 310009 Zhejiang China; 2 Department of orthopedic Surgery, The Second Affiliated Hospital, School of Medicine, Zhejiang University, Hangzhou, 310009 Zhejiang China; 3 Departments of Anesthesiology, The Third Affiliated Hospital of Xinjiang Medical University, Ürümqi, 830011 Xinjiang China; 4 Cancer Institute (Key Laboratory of Cancer Prevention and Intervention, China National Ministry of Education), The Second Affiliated Hospital, School of Medicine, Zhejiang University, Hangzhou, 310009 Zhejiang, China; 5 Departments of Pulmonary Medicine, The Third Affiliated Hospital of Xinjiang Medical University, Ürümqi, 830011 Xinjiang China

## Abstract

Osteosarcoma (OS) is the most common primary malignant bone tumor mainly occurring in children and adolescents. In past decades, studies revealed that *PARK2* was a vital tumor suppressor gene in many malignant solid tumors. However, the role of *PARK2* in OS remains largely unclear. Therefore, we assessed PARK2 expression in OS tissue and adjacent non-tumor tissues by immunohistochemical (IHC) analysis, and evaluated PARK2 mRNA expression in OS cell lines by real-time PCR analysis. The HOS and U2OS cell lines were employed to establish a *PARK2* overexpression model. Using this model, we investigated the potential role of *PARK2* in OS and explored the underlying molecular mechanisms. Our study showed PARK2 was downregulated in OS tissue and cell lines, which was significantly associated with higher tumor stage (*P* < 0.05). Overexpression of *PARK2* arrested the cell cycle, inhibited cell proliferation, migration, and invasion, induced cell apoptosis, and reduced tube formation in vitro. Moreover, overexpression of *PARK2* significantly suppressed tumor growth and angiogenesis in vivo. Additionally, *PARK2* negatively regulated OS development through the JAK2/STAT3/VEGF pathway. Our findings demonstrate that *PARK2* is a tumor suppressor gene that may negatively affect OS growth and angiogenesis via partly inhibiting the JAK2/STAT3/VEGF signaling pathway.

## Introduction

Osteosarcoma (OS) is the most common primary malignant bone tumor that mainly occurs in children and adolescents^1–4^. OS is usually located in the metaphysis of long bones, especially near the knee^5^. The incidence rate is approximately four people per million each year^6,7^. Combined surgical resection and intensive chemotherapy has improved the 5-year overall survival rate (from 51 to 75%)^6–11^. However, the 10-year survival rate and long-term free survival rate remain unsatisfactory (50% or less)^10^. These poor survival rates may be due to the high metastatic rate. That is, 13% of patients had distant metastases at the time of diagnosis^11^, and more than 30% develop distant metastases after treatment^12^. Thus, understanding OS pathogenesisis crucial in managing this lethal, highly metastatic disease.

PARK2 is widely expressed in various tissues and encodes an E3 ubiquitin ligase for proteosome-mediated protein degradation^13^. Veeriah et al. identified *PARK2* as a frequently targeted gene on chromosome 6q25.2–q27^14^. This region is known to be unstable and prone to breakage and rearrangement^15,16^, with ~500 breakpoint junctions involving *PARK2*^17^. Previous reports have shown that deletions of *PARK2* occurin 30% of human malignant tumors^18^, including glioma, breast, liver, lung, pancreatic, and colorectal cancers^19–24^. *PARK2* deletion or mutation directly eliminates or reduces PARK2 protein production in cells, respectively, and improves tumor growth in vitro and vivo^21–23^. In this regard, *PARK2* is a potential candidate tumor suppressor gene, because when deleted or mutated, it can allow cells to grow uncontrollably with enhanced tumor formation. However, the role of PARK2 in OS remains unclear. Therefore, we hypothesized that *PARK2* gene overexpression can inhibit tumorigenesis in OS.

PARK2 deficiency enhances tumor cell proliferation^19–23^, increases the resistance to apoptosis^21^, and promotes tumor development in vivo^19,20,23^. Previous studies have shown that PARK2 negatively regulates the biological function of malignant tumors through several signaling pathways, including the Wnt, EGFR–AKT^20^, and PI3K/AKT/mTOR^25^ pathways. Notably, the Janus Kinase 2 (JAK2)/Signal Transducer Activator of Transcription 3 (STAT3)/vascular endothelial growth factor (VEGF) signaling pathway has been associated with many solid tumors^26^. This pathway participates in regulating tumor angiogenesis, which plays a pivotal role in the growth, invasion, and metastasis of various malignant tumors, including OS^27^. Whether the JAK2/STAT3/VEGF pathway is also associated with the *PARK2* gene remains unknown.

In the current study, we aimed to determine whether the *PARK2* gene is related to OS growth, metastases, and angiogenesis. We also ascertained whether PARK2 is involved in regulating the expression of VEGF by inhibiting the JAK2/STAT3 pathway. Moreover, we observed the changes in expression of the VEGF, p-JAK2, and p-STAT3 proteins using interleukin-6 (IL-6) and stattic interference of the JAK2/STAT3 signaling pathway activation in OS cells.

## Results

### PARK2 is downregulated in OS tissue and cell lines

**Fig. 1:**
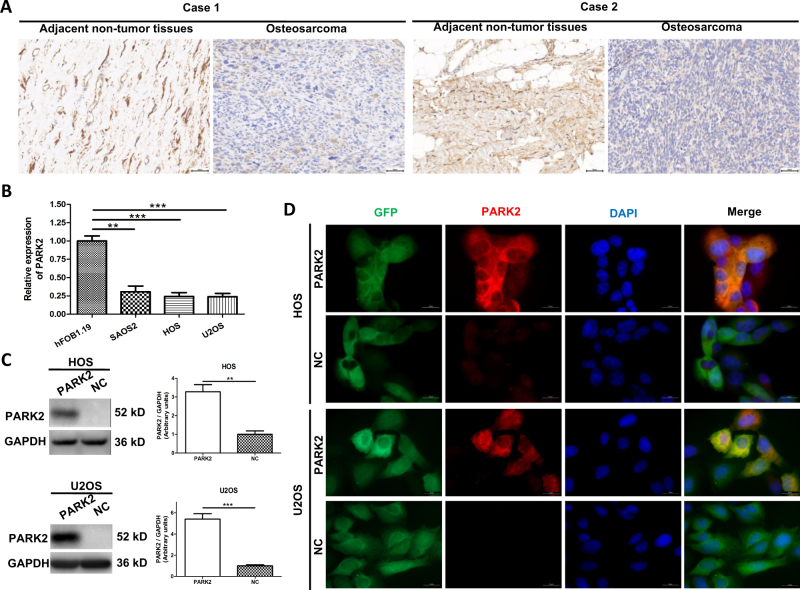
PARK2 is downregulated in osteosarcoma tissues and cell lines, and PARK2 overexpression models are constructed. There are two representative cases comparing OS tissues and adjacent non-tumor tissues detected by immunohistochemistry (**a**). The expression of PARK2 mRNA in SAOS2, HOS, U2OS, and hFOB1.19 cells (**b**). The expression level of the protein Parkin was significantly enhanced in HOS and U2OS cell lines after transfection was detected using western blot assays (**c**) and immunofluorescence assays (**d**). Magnification, ×200 (**a**), ×1000 (**d**). Scale bar, 50 μm (**a**, **d**). **P < 0.01, ***P < 0.001

To evaluate the role played by PARK2 in OS development, 46 primary OS tissues and their adjacent non-tumor tissues were studied using PARK2 IHC (Fig. 1a). The results showed that 76% (35/46) of the adjacent non-tumor tissues and 37% (17/46) of the OS tissues expressed the PARK2 protein (*P* < 0.05). Moreover, the expression of PARK2 was significantly associated with higher tumor stage (*P* < 0.05, Table 1). Next, we examined PARK2 mRNA expression by real-time PCR in the human OS cell lines SAOS2, HOS, and U2OS, and in the normal human osteoblastic cell line hFOB1.19. The level of PARK2 mRNA decreased significantly in the OS cell lines (Fig. 1b). Collectively, these results demonstrate that PARK2 is downregulated in human OS tissues and cells, and it is associated with OS progression, which might play a role in suppressing human OS development.

**Table 1:** Clinicopathologic features and the expression status of PARK2 in 46 osteosarcoma patients

	Parameter	Case	PARK2 expression		*P* value
			Positive	Negative	
Age (year)	<18	14	6	8	0.742
	≥18	32	11	21	
Sex	Male	30	10	20	0.534
	Female	16	7	9	
Histological grade	Well and moderately differentiated	9	6	3	0.255
	Poorly differentiated	37	11	26	
Tumor	T1	27	13	14	0.067
	T2	19	4	16	
Stage	I–II	39	17	22	0.036
	III	7	0	7	

### PARK2 overexpression inhibits OS cell proliferation in vitro

**Fig. 2:**
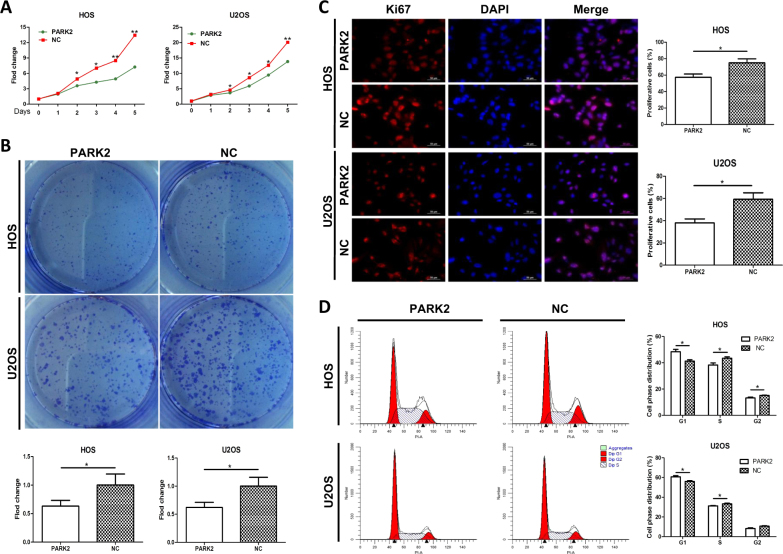
Overexpression of PARK2 inhibits osteosarcoma cell proliferation in vitro. PARK2 significantly inhibits cell proliferation (**a**) and colony formation (**b**) compared with NC in HOS and U2OS cell lines. Compared with NC, PARK2 downregulated the cell proliferation rate (**c**) and arrested the progression of the cell cycle (**d**) in HOS and U2OS cell lines. These data were detected by an immunofluorescence assay and flow cytometry, respectively. Magnification, ×400 (**c**). Scale bar, 50 μm (**c**). *P < 0.05, **P < 0.01.Each assay was repeated three times

PARK2 shows low expression levels in the HOS and U2OS cell lines, which were selected for overexpression models. Transfection rates of *PARK2* gene overexpression group (HOS-PARK2 and U2OS-PARK2) and negative control group (HOS-NC and U2OS-NC) were close to 90%, which were further confirmed by western blot and immunofluorescence assay (Fig. 1c, d). The stably transfected cells were used to investigate biological functions and potential mechanisms in OS. Cell viability (Fig. 2a) and colony formation assays (Fig. 2b) showed that the PARK2 group significantly inhibited cell growth relative to that in the NC group (*P* < 0.05). Ki67 staining revealed cell proliferation rates in PARK2 and the NC group were 57.33% ± 4.06% vs. 75.03% ± 4.67% in HOS cells and 38 ± 3.61% vs. 59.33 ± 5.81% in U2OS cells, respectively (*P* < 0.05, Fig. 2c). Cell cycle analysis revealed significantly decreased cell percentages in the G2/M phase (13.25% ± 0.60 and 8.27% ± 0.68%) and S phase (38.33 ± 1.56% and 31.16 ± 0.50%) for PARK2 relative to those of the NC group (G2/M phase: 15.19 ± 0.21% and 10.96 ± 0.80%; S phase: 43.52 ± 1.02% and 34.32 ± 0.77%) in the HOS and U2OS cell lines, respectively (Fig. 2d). These results indicate that the cell cycle was arrested by the *PARK2* gene.

### PARK2 overexpression suppresses OS cell migration and invasion

**Fig. 3:**
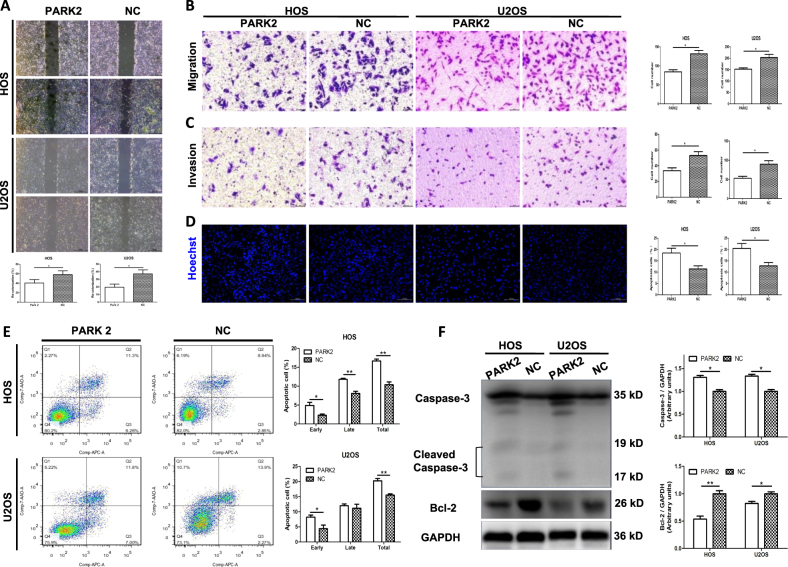
Overexpression of PARK2 inhibits osteosarcoma cell migration and invasion and promotes osteosarcoma cell apoptosis in vitro. The wound-healing assay showed that the area of co-colonization in the PARK2 group was lower than the NC group (**a**). The migration (**b**) and invasion (**c**) ability of HOS and U2OS cells were significantly attenuated in the PARK2 group relative to the NC group, which was evaluated using a Transwell assay. An immunofluorescence assay showed that the ratios of karyopycnosis, nuclear fragmentation, and apoptotic body formation in the PARK2 group were greater than in the NC group (**d**). A flow cytometry assay indicated that the percent of total apoptotic cells significantly increased in the PARK2 group (**e**). A western blot assay revealed the level of protein caspase-3 and cleaved caspase-3 increased and the level of protein bcl-2 decreased in the PARK2 group compared with the NC group (**f**). Magnification, ×40 (**a**), ×200 (**b**, **c**), ×200 (**d**). Scale bar, 500 μm (**a**), 200 μm (**b**, **c**), 100 μm (**d**). *P < 0.05, **P < 0.01, ***P < 0.001. Each assay was repeated three times

Using a wound-healing assay, we evaluated the effect of the *PARK2* gene on OS cell migratory behavior, a critical determinant of metastasis in tumor progression. Compared with the NC group, the PARK2 groups significantly decreased the cell re-colonization into the wound area after 24 h in HOS and U2OS cell lines (*P* < 0.05, Fig. 3a). In the Transwell chamber analysis, the *PARK2* gene reduced the ability for OS cells to migrate with respect to that in the NC group after 12 h (HOS: 84.33 ± 5.61 vs. 131.7 ± 8.95 cells per field; U2OS: 152.30 ± 5.78 vs. 203.00 ± 14.11 cells per field; *P* < 0.05, Fig. 3b). A Transwell chamber assay showed that *PARK2* overexpression significantly suppressed cell invasion in OS cells 24 h after treatment (HOS: 33.67 ± 3.71 vs. 53.00 ± 5.03 cells per field; U2OS: 53.00 ± 5.14 vs. 89.67 ± 9.21 cells per field; *P* < 0.05, Fig. 3c).

### PARK2 overexpression promotes OS cell apoptosis

Karyopycnosis, nuclear fragmentation, and apoptotic body formation were detected with Hoechst (Beyotime, China) staining. The positive nuclear expression of PARK2 was significantly increased compared with the NC cells (*P* < 0.05, Fig. 3d). Cell apoptosis was also detected using a flow cytometry assay after annexin V-APC/7-AAD (MultiSciences, China) double staining. In the HOS cell line, ~4.90 ± 0.79% of cells showed early apoptotic properties among the PARK2 cells. This percentage was significantly higher than that among the NC cells (2.35% ± 0.31%, *P* < 0.05). The total apoptotic cell percentage was also higher in the PARK2 cells than in the NC cells (*P* < 0.05). We obtained similar results for the U2OS cell lines (Fig. 3e). A western blot was used to test the apoptosis-related protein expression changes after *PARK2* overexpression in the HOS and U2OS cell lines (Fig. 3f). Quantitative analysis of the blot intensities revealed that the *PARK2* gene significantly increased caspase-3 and cleaved caspase-3 protein levels, but decreased Bcl-2 protein levels, compared with the NC cells in the OS cells (*P* < 0.05).

### PARK2 overexpression downregulates OS tumor growth in vivo

**Fig. 4:**
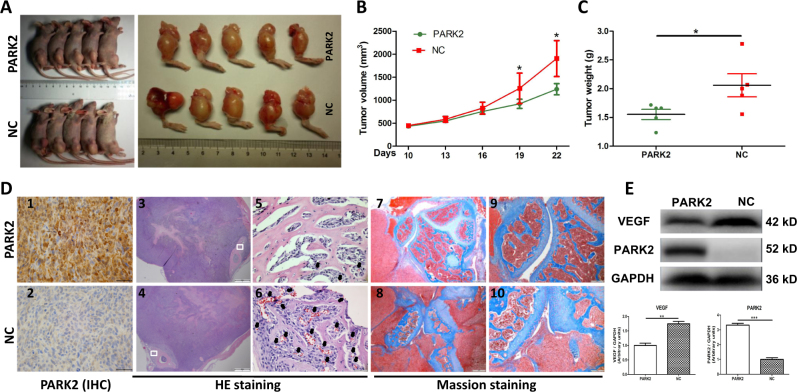
Overexpression of PARK2 inhibits osteosarcoma growth in vivo. The photograph shows tibial tumor formation in two groups (**a**). Compared with the NC group, PARK2 significantly decreased tumor size and tumor weight by the end of the experiment (**b**, **c**). PARK2 expression in tibial xenografts was confirmed by immunohistochemistry (IHC) (**d**1–2). Histological characteristics were evaluated by H&E staining; extensive tumor cell necrosis, numerous hematocytes, and vascular structures (black arrows) were observed in the NC group (**d**3–6). Joint structure destruction was evaluated by Masson staining (**d**7–10). The western blot assay showed the expression level of the proteins VEGF and PARK2 in tibial xenografts in two groups that the expression of the protein VEGF was negatively correlated with PARK2 (**e**). Magnification, ×20 (**d**3–4), ×40 (**d**7–8), ×100 (**d**9–10), ×400 (**d**1–2, 5–6). Scale bar, 1000 μm (**d**3–4), 500 μm (**d**7–8), 200 μm (**d**9–10), 50 μm (**d**1–2, 5–6). *P < 0.05, **P < 0.01, ***P < 0.001

In vitro studies have shown that PARK2 suppressed OS cell proliferation, migration, and invasion. Further study confirmed the potential effect of the *PARK2* gene on OS formation in vivo. HOS stably transfected cells were employed for tibial tumor formation. The data showed that the *PARK2* gene significantly inhibited tumor growth in vivo (Fig. 4a); at the end of the experiment, the tumor sizes were 1238 ± 121.8 mm^3^ vs. 1,905 ± 390.8 mm^3^ and the tumor weights were 1.552 ± 0.088 g vs. 2.058 ± 0.201 g in the PARK2 and NC groups, respectively (*P* < 0.05, Fig. 4b, c). Tumor specimens were used for further pathological analysis. IHC detection showed that the PARK2 protein level in the PARK2 group was dramatically higher than that in the NC group (Fig. 4c1, 2). Hematoxylin and eosin (H&E) staining revealed that the PARK2 group attained fewer hemocytes and vascular structures in the Codman-triangle area than those in the NC group (Fig. 4d3–6). Masson staining showed that the knee joint structure of the PARK2 group was intact; the joint cavity exhibited no obvious narrowing, and the articular surface was smooth (Fig. 4d7–10). However, in the NC group, the OS cells invaded the articular cavity, the articular cavity narrowed, and the articular cartilage of the tibia was severely damaged (Fig. 4d10). The western blot assay revealed that the *PARK2* gene was negatively associated with the expression level of the protein VEGF in the tibial xenografts (*P* < 0.05; Fig. 4e).

### PARK2 overexpression downregulates the JAK2/STAT3/VEGF signaling pathways

**Fig. 5:**
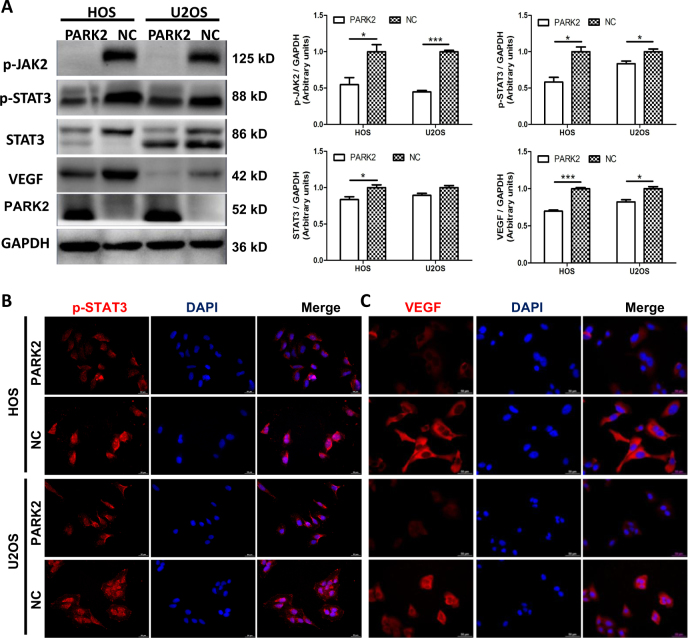
PARK2 regulates osteosarcoma cell growth and metastases by suppressing the Jak2/STAT3/VEGF signaling pathway. The western blot assay indicated that the level of proteins p-JAK2, p-STAT3, and VEGF in the PARK2 group was significantly lower than that in the NC group. Representative images (left) and quantification (right) of the western blot assay (**a**). The immunofluorescence assay showed the expression of the proteins p-STAT3 and VEGF in the HOS and U2OS cell lines (**b**, **c**). Magnification, × 400 (**b**, **c**). Scale bar, 50 μm (**b**, **c**). * *P* < 0.05, ** *P* < 0.01, *** *P* < 0.001. Each assay was repeated three times

**Fig. 6:**
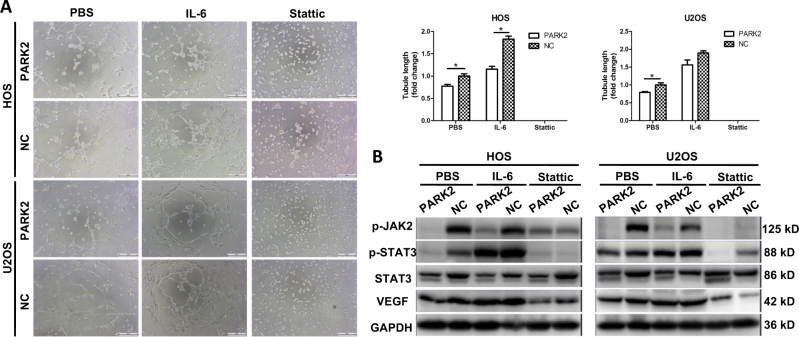
Overexpression of PARK2 inhibits osteosarcoma cell tube formation in vitro. NC was used as the control group. The formation of capillary-like structures was obviously suppressed by PARK2. The co-culture with IL-6 (25 ng/ml) or stattic (10 μM) was also evaluated (**a**). The western blot analysis of the expression of the proteins p-Jak2, p-STAT3, STAT3, and VEGF post co-culture with IL-6 (25 ng/ml, 30 min) or stattic (10 μM, 2 h) (**b**). Magnification, ×100 (**a**). Scale bar, 200 μm (**a**). *P < 0.05, **P < 0.01, ***P < 0.001. Each assay was repeated three times

The tibial implants tumor showed that *PARK2* reduced tumor angiogenesis and the amount of the VEGF protein. We speculated that PARK2 is associated with the JAK2/STAT3 pathway in OS. Notably, *PARK2* overexpression significantly decreased the levels of p-JAK2 and p-STAT3 proteins (*P* < 0.05, Fig. 5a). Meanwhile, the level of the protein VEGF in the PARK2 group was significantly lower than that in the NC group (*P* < 0.05, Fig. 5a). Fluorescence microscopy was utilized to examine the p-STAT3 and VEGF localization and relative expression intensity in the PARK2 and NC cells because the nuclear localization and high expression of these proteins were correlated with their activities. Compared with the NC cells, there was less p-STAT3 protein located in the cell nucleus, and reduced p-STAT3 and VEGF fluorescence intensity in the *PARK2* overexpression cells (Fig. 5b, c). Moreover, interleukin-6 (IL-6: 25 ng/ml, 30 min) and stattic (10 μM, 2 h), the p-STAT3 agonist and an inhibitor, respectively, were used to activate or inhibit STAT3 phosphorylation. The results indicated that the level of p-JAK2 and p-STAT3 were significantly reduced in PARK2 cells compared with NC cells after incubation with IL-6 and stattic (Fig. 6b).

### PARK2 overexpression inhibits tube formation in vitro

VEGF is known to play a critical role in angiogenesis. We further investigated the effects of the *PARK2* gene on tubule formation in vitro. Capillary-like structures were formed in the HOS and U2OS cell lines (Fig. 6a). The tube length in the PARK2 group was significantly shorter than that in the NC group (*P* < 0.05). Recombinant IL-6 (25 ng/mL) significantly increased tubule formation in the two groups. The difference in the tube length was significant in the HOS cell line (*P* < 0.05), but there were no differences between the PARK2 and NC groups in the U2OS cell line. However, tubule formation ability was blocked by stattic (10 μM), and no difference was observed in HOS and U2OS cell lines (*P* > 0.05).

**Fig. 7:**
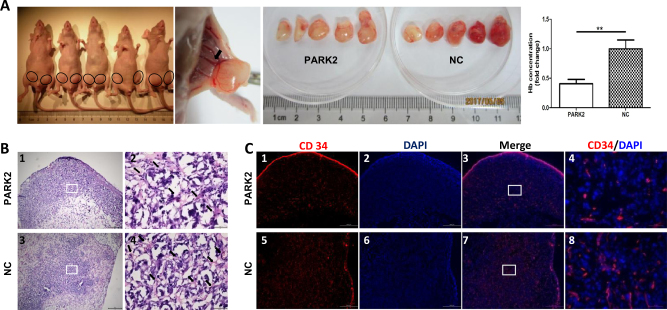
Overexpression of PARK2 decreases angiogenesis in vivo. Nude mice were injected on both sides of the groin subcutaneously with Matrigel mixed with the stable transfected HOS cells (8 × 10^6^ cells; PARK2 on the right side; NC on the left side) and were left for 7 days. General morphology and color of the Matrigel plugs were recorded and plugs were quantified for hemoglobin content (**a**). Hematocytes and vascular structures (black arrows) were detected by H&E staining (**b**). Vascular endothelial-like structures were examined by a CD34 immunofluorescence staining assay (**c**). Magnification, ×40 ((**b**1, 3), (**c**1–3, 5–7)), ×400 ((**b**2, 4), (**c**4, 8)). Scale bar, 500 μm ((**b**1, 3), (**c**1–3, 5–7)) and 50 μm ((**b**2, 4), (**c**4, 8)). *P < 0.05, **P < 0.01, ***P < 0.001

PARK2 overexpression decreases angiogenesis in vivo PARK2-mediated angiogenesis was further confirmed by the in vivo Matrigel plug assay. We found that the inguinal vessels sent branches into the Matrigel plugs (Fig. 7a). Under further assessment of the angiogenesis level by measuring hemoglobin concentrations in the Matrigel plug, we found that PARK2 decreased hemoglobin levels by 40.65 ± 7.51% with respect to that in the NC (*P* < 0.05). H&E staining (Fig. 7b) and immunofluorescence staining of CD34 (Fig. 7c) showed that PARK2 reduced the number of hematocytes in the plugs and decreased the tumor microvessel density in the Matrigel plugs.

## Discussion

The *PARK2* gene was first isolated from an autosomal recessive juvenile parkinsonism patient. The gene is located at the long arm of chromosome 6 (6q25.2–q27) and encodes a protein with 465 amino acids with moderate similarity to ubiquitin at the amino terminus^28,29^. The loss of heterozygosity within the chromosomal region 6q25–q27 is frequently associated with various solid tumor types^21^. In the past two decades, PARK2 has been found to encode an E3 ubiquitin ligase, which is widespread in human tissues and 12 other species^29^. Since then, the biological functions of PARK2 have been gradually identified. PARK2 has been shown to be a causal factor of autosomal recessive juvenile Parkinson’s disease and is involved in mitochondrial homeostasis, energy metabolism, xenophagy, protein turnover, stress response, and regulated cell growth and survival^30–32^. *PARK2* deletion, which leads to growth free from suppression, is uncontrolled in various cancer cell lines and promotes cancer cell proliferation in vitro^19–21,33^. Clinical studies also have shown that low PARK2 transcription correlates with increased lymph node metastasis, increased tumor grade, and worsened overall survival in malignant tumors^30,33,34^. Our study shows that PARK2 has a low expression in OS tissue and cell lines, and is associated with tumor stage. These data strongly suggest that *PARK2* is a tumor suppressor gene in OS.

Evidence suggests that PARK2 plays an anti-oncogenic role in malignant tumor development because it inhibits tumor cell growth, impedes metastasis, and promotes apoptosis in vitro and in vivo^19–24^. Tay et al. reported that PARK2 significantly increased the expression level of cyclin-dependent kinase 6 (CDK6) in breast cancer cells, which consequently downregulated the cell cycle and proliferation^19^. Gong et al. also demonstrated that PARK2 targets both cyclin D and cyclin E for degradation; the two cyclins regulate the stability of G1/S cyclins^35^. *PARK2* overexpression accelerates the arrest of the G1-phase and delays the entry to mitosis^19,30,33^. Consistent with these findings, stably transfected OS cells were detected in the cell proliferation assay, colony formation assay, and cell cycle assay. The results revealed that PARK2 reduced the OS cell proliferation rate and colony forming efficiency by arresting more OS cells at the G1 phase and blocking cell entry into the S and G2/M phase in vitro. Tumor formation in nude mice further confirmed that OS growth was significantly suppressed by the PARK2 gene in vivo. These results suggested that *PARK2* was an important anti-oncogene, and its overexpression/deficiency is associated with OS development.

In general, cell pro- and anti-apoptosis is in a state of balance. Impaired apoptotic ability is an essential characteristic of tumor cells. Mitochondria are the main site for aerobic respiration and apoptotic regulation. Cytochrome c release and crossing of the outer membrane is a crucial step in the activation of the caspase cascade. Whether PARK2 is pro- or anti-apoptosis remains to be debated. Darios et al. reported that PARK2 alters the delay of mitochondrial swelling, cytochrome c release, and caspase-3 activation^36^. Another study showed that the effect of PARK2 on mitochondrial mechanisms governing cytochrome c release and apoptosis is relevant to selective neuronal vulnerability^37^. These results suggest that PARK2 protects cells against apoptotic stress. However, studies on tumor cells show that PARK2 promotes tumor cell apoptosis in breast cancer^38^. *PARK2* deficiency also resulted in the suppression of caspase activation and rendered hepatocytes resistant to apoptosis^21^. Recently, Gong et al. reported that PARK2 directly binds and ubiquitinates BCL-XL, which regulates mitochondrial outer membrane permeability and apoptosis^39^. In the present study, the Hoechst staining assay and flow cytometry assay showed that additional apoptotic cells were induced in *PARK2* overexpression cells. The western blotting assay indicated that PARK2 rescued the caspase-3 protein, cleaved caspase-3 expression, and decreased the expression level of the protein BCL-2 in OS. Thereby, we confirmed the conclusion that PARK2 was pro-apoptosis in tumor cells.

Angiogenesis is known as the formation of new blood vessels from existing vasculature. The process is employed by virtually all malignant solid tumors to initiate and facilitate tumor progression^40,41^. The ability to activate or inactivate this angiogenic switch is considered a hallmark of maligntumors^42^. The ability to inhibit angiogenesis is a mechanism that blocks cell growth and decreases cell metastasis. In the current study, PARK2 suppressed the growth of OS cells and also significantly decreased OS cell migration and invasion in vitro. Interestingly, tibial xenograft section analysis revealed that fewer hematocytes and microvessel-like structures were detected in the PARK2 group at the Codman-triangle area than in the NC group. A tube formation assay was carried out to confirm the angiogenic potential of OS cells. The data showed that PARK2 reduced the formation of the capillary-like structures in vitro and the tubule formation significantly activated by IL-6 or inhibited by stattic in vitro. PARK2 also decreased neovascularization and tumor microvessel density in vivo. These results suggested that *PARK2* is a potential anti-angiogenesis gene that can downregulate OS cell migration, invasion, and metastasis. Hence, *PARK2* mutation/loss may also be involved in OS progression through the regulation of OS angiogenesis. However, numerous studies propose that angiogenesis is directly correlated with the JAK2/STAT3 signaling pathway.

Finally, we further explored the underlying mechanism by which PARK2-related signaling pathways inhibits OS angiogenesis and negatively affects OS cell growth, migration, and invasion. Previous studies have shown that JAK2/STAT3 signaling regulates several important pathways in tumorigenesis, including cell cycle progression, apoptosis, tumor invasion, and metastasis^43,44^. Thus, the gene is a potential therapeutic target in various human solid tumors^26,43^. VEGF is a downstream protein of the JAK2/STAT3 signaling pathways that plays a pivotal role in angiogenesis^45,46^. VEGF is usually upregulated in multiple cancers, including OS^46^. To investigate whether the suppression or inactivation of this pathway is inherently associated with the *PARK2* gene, the stably overexpressed PARK2 cell lines were used. Cells with *PARK2* overexpression exhibited a pronounced decrease in JAK2 and STAT3 phosphorylation relative to that in the NC cells (Fig. 5). The downstream protein of VEGF was also downregulated by PARK2 in the OS cell lines and tibial xenografts (Fig. 4e). To further investigate the mechanisms of PARK2, we activated or inhibited STAT3 phosphorylation using its agonist (IL-6, 25 ng/ml) and inhibitor (stattic, 10 μM) in the in vitro model (Fig. 6). These results show that the expression of the VEGF protein, in parallel with p-JAK2 and p-STAT3 expression, in the OS cells was decreased by PARK2. Accordingly, we propose that PARK2 inhibits the proliferation, migration, invasion, angiogenesis, and metastasis of OS via partly downregulating the JAK2/STAT3/VEGF signaling pathway.

PARK2 can inhibit the JAK2/STAT3/VEGF signaling pathway, and also through association with other genes and protein–protein interactions, regulate the network of growth pathways, thus becoming a vital tumor suppressor. Recently, a study conducted by Gupta et al. indicated that PARK2 depletion promotes PTEN inactivation^25^. The inactivation of the *PTEN* gene has been found in many of human cancer cell lines and primary tumors, and is ranked the most mutated tumor suppressor gene after p53^47^. PTEN mutation and subsequent activation of PI3K/Akt signaling promotes the activation of mTOR signaling, which in turn promotes cancer cell survival, growth, cell cycle progression, angiogenesis, protein synthesis, and other cellular processes^47,48^. Therefore, the overexpression of *PARK2* in OS cells leads to cell survival, growth, metastasis, and angiogenesis could be partly inhibited by the downregulated PI3K/Akt and mTOR signaling pathways too.

This study holds certain limitations, as follows. (1) The expression of PARK2 was assessed in 46 human OS tissue specimen by IHC analysis. Larger sample sizes are needed in the future. (2) The expression levels of PARK2 mRNA or proteins were fairly low in the OS cell lines. Thus, no knockdown or knockout models were evaluated in this study, so additional information is required. This limited experimental approach does not comprehensively explain the biological functions and molecular mechanisms of the *PARK2* gene in OS. However, as an important tumor suppressor in OS, we have provided limited but valuable information on the relationship of *PARK2* and OS.

The current study demonstrated that *PARK2* is a tumor suppressor gene in OS and exerts a profound anti-cancer activity in vitro and in vivo, including retarding growth, migration, invasion, metastasis, and angiogenesis by partly inhibiting the JAKT2/STAT3/VEGF pathway. These findings increase the understanding of OS progression, and *PARK2* may be applied as a prognostic indicator for OS.

## Materials and methods

### OS tissue samples and cell culture

Forty-six pairs of paraffin-embedded sections were obtained from the Department of Pathology at the Second Affiliated Hospital of Zhejiang University, School of Medicine. All specimens were obtained with informed consent from the patients. This study was approved by the Ethics Committee of the Second Affiliated Hospital of Zhejiang University.

SAOS2, HOS, and U2OS, and the normal human osteoblastic cell line hFOB1.19 were purchased from the Cell Bank of the Chinese Academy of Sciences (Shanghai, China). SAOS2 and HOS cells were cultured in Dulbecco’s modified Eagle’s medium (DMEM) containing 10% fetal bovine serum (FBS) and 1% penicillin/streptomycin at 37 °C in an environment with 5% CO_2_. U2OS cells were cultured in RPMI 1640 containing 10% FBS and 1% penicillin/streptomycin at 37 °C with 5% CO_2_.The hFOB1.19 cells were cultured in DMEM/Ham’sF-12 supplemented with Geneticin (400 mg/mL) and 10% FBS at 33.5 °C with 5% CO_2_. The cells were harvested and subcultured upon reaching 80–90% confluence.

### Immunohistochemistry (IHC) and analysis

The paraffin-embedded sections were de-waxed in xylene and rehydrated in ethanol. Then, sections were performed using heat-induced epitope retrieval in 0.01 M citrate buffer at pH 6.0. Sections were washed three times with PBS, blocked with 5% BSA, and the sections were incubated with primary antibody (PARK2, Abcam, USA) overnight at 4 °C.Then, the sections were incubated with secondary antibody (Biological Technology, China) for 1 h at room temperature, stained with the enzyme substrate 3′,3-diaminobenzidinetetrahy-drochloride (DAB, Biological Technology, China) and counterstained following the manufacturer’s instructions.

The staining intensity and the percentage of stained cells were measured to evaluate PARK2 expression in IHC. The staining intensity of PARK2 was scored as 0 (negative), 1 (weak), 2 (moderate), or 3 (strong), and the percentage of positively stained cells was also scored as 0 (≤5%), 1 (6–25%), 2 (26–50%), 3 (51–75%), 4 (>75%). The product of the staining intensity and percentage composed the final staining score (0–12), and the scores were converted into sum indices (−, score 0; + , score ~1–4; + + , score ~5–8, and + + + , score ~9–12). Each section was independently analyzed by two pathologists.

**qRT-PCR** Total RNA from the SAOS2, HOS, U2OS, and hFOB1.19 cells was extracted using TRIzol reagent (Invitrogen, Carlsbad, CA, USA). Relative expression levels of PARK2 mRNA were detected by real-time PCR on a StepOnePlus Real-Time PCR system (Life Technologies, Foster, CA, USA). The relative expression of mRNA was described as 2^−ΔCt^(ΔCt = Ct_PARK2_ − Ct_GAPDH_). The following primers were used: PARK2, forward, 5′-ATCGCAACAAATAGTCGG-3′ Reverse: 5′-GGCAGGGAGTAGCCAAGT-3′; GAPDH, forward, 5′-TCGACAGTCAGCCGCATCT-3′ Reverse: 5′-CTTGACGGTGCCATGGAATT-3′.

### Constructing stable overexpression cell lines (transfection)

A *PARK2* gene overexpression (PARK2) plasmid and negative control (NC) plasmid were purchased from Genepharma corporation (Shanghai, China). All the procedures were based on the manufacture protocols. GFP expression was observed under a fluorescence microscope after 24 h. The transfected cells were incubated with 2 μg/mL puromycin until all the cells died in the control group (untransfected cells). The transfection efficiency of the cells was further confirmed by immunofluorescence staining and western blot before further analysis.

### Cell proliferation assays

To test the proliferation effects of PARK2, transfected cells were seeded into a 96-well plate at 2 × 10^3^ cells per well. After 6 h, a CCK8 Kit (Dojindo, Japan) was used to quantitatively analyze cell viability. CCK8 reagent (10 μL) was added to each well, and after incubation for 2 h, the plates were detected using a microplate reader at 450 nm absorbance daily for 6 days. Colony formation was used to evaluate the cell proliferation activity. Cells in the exponential growth phase were seeded onto the 6-well plate. The HOS and U2OS cell densities were 1,000 and 500 cells per well, respectively. The cells were incubated for 14–21 days, and the colonies were visualized under a microscope and photographed after crystal violet staining. The number of colonies (>50 cells/colony) was recorded.

### Cell invasion and migration assays

A wound-healing assay was utilized to detect the cell migration capacity. Under microscopic observations, cell proliferation reached about 90% confluence. A pipette tip was used to scratch the cell layer firmly to make a wound line. Then, the cells were washed a few times with PBS to remove debris, after which serum-free medium was added. The cells were visualized and photographed under a microscope immediately and after 24 h. The wound area was analyzed and compared using the software ImageJ.

Transwell chambers (with 8 μm-pore-sized membranes, Corning Life Science, USA) with Matrigel (for invasion assay) and without Matrigel (for migration assay) were used to further analyze the cell invasion and migration abilities. First, 5 × 10^4^ cells were seeded in the upper chambers with serum-free medium, and cells were seeded in the bottom chambers with medium containing 10% fetal bovine serum. After 8 h of incubation, the membranes were drenched in 4% paraformaldehyde for 15 min and subsequently stained with crystal violet for 30 min. Then, cells were wiped off the upper membrane. The migrating or invading cells were visualized by inverted microscopy.

### Flow cytometry assays

The transfected cells were seeded onto a 6-well plate at a density of ~1 × 10^5^ cells per well. After 24 h, the cells were harvested with trypsin and washed three times with chilled PBS buffer. The cells were then stained with Annexin V-APC/7-aminoactinomycin D (7-AAD; MultiSciences, China) at room temperature in the dark. All procedures followed the manufacturer’s protocols. Then, an FAC Scan flow cytometer (Becton–Dickinson, USA) was used to analyze the samples and measure the percentage of apoptotic cells.

Cell cycle analysis was also used to further evaluate cell proliferation. Briefly, cells were fixed with chilled 70% ethanol and stored at −20 °C overnight. The next day, the cells were centrifuged after the supernatant was discarded, rewarmed, and stained with PI (50 μg/mL; MultiSciences, China) for 30 min before flow cytometry. More than 3 × 10^4^ cells were detected.

### Western blot assays

After proper treatment, the cells and tissues were lysed in RIPA buffer (Sigma, USA) with a protease inhibitor and phosphatase inhibitor cocktail. The same amounts of protein samples were separated by SDS–PAGE and transferred onto a polyvinylidene fluoride membrane. After blocking with 10% milk or 3% BSA, the membranes were incubated with primary antibodies overnight at 4 °C and then with horseradish peroxidase (HRP)-conjugated secondary antibody for 1 h at room temperature. Concentrations adopted for the above-mentioned antibodies were based on the manufacturers’ specifications. Finally, the membranes were observed using an enhanced chemi-luminescence kit (Millipore, Bedford, MA). Blot intensity was analyzed with Quantity One. The details for the primary antibodies were as follows: Parkin(1:1000, Cat: ab15954, Abcam), p-STAT3 (1:100000, Cat: ab76315, Abcam), Caspase-3 (1:1000, Cat: 9662 s, Cst), Bcl-2 (1:1000, Cat: 15071 s, Cst), STAT3 (1:1000, Cat: 12640 s, Cst), p-JAK2 (1:1000, Cat: 3776 s, Cst), VEGF (1:1000, Cat: RT1649, Huabio), and GAPDH (Cat: RT1210-1, Huabio). The secondary antibodies were as follows: goat anti-rabbit IgG–HRP (1:2000, Cat: HA1001-100, Huabio) and goat anti-mouse IgG–HRP (1:2000, Cat: HA1006, Huabio).

### Immunofluorescence

Immunofluorescent staining was performed to assess the relative protein expression of cell lines and tissues. Each sample was fixed with 4% paraformaldehyde at room temperature. After blocking with endogenous peroxidase, the samples were permeabilized and blocked with 3% BSA for 1 h, then incubated with primary antibody at 4 °C overnight, and secondary antibody was used at a 1:500 dilution in 3% BSA and incubated at room temperature for 1 h. The samples not incubated with primary antibody were designated as negative controls. These cells or tissues were observed and photographed under a fluorescence microscope. The details of the primary antibodies were as follows: Parkin (1:100, Cat: ET1702-60, Huabio), p-STAT3 (1:500, Cat: ab76315, Abcam), CD34 (1:200, Cat: ab81289, Abcam), and VEGF (1:200, Cat: RT1649, Huabio). Secondary antibodies included goat anti-rabbit (1:200, Cat: HA1004, Huabio) and goat anti-mouse (1:200, Cat: HA1003, Huabio) antibodies.

### In vivo tumor formation assay

All experiments were performed in accordance with the guidelines of the Zhejiang University Ethics Committee. The study was approved by the Medical Ethics Committee to observe the formation and metastasis of OS in vivo. Four-week-old nude mice were randomized into PARK2 and NC groups (*n* = 10), and about 2 × 10^6^ stable transfected cells were injected into the marrow cavity of the right tibia to establish a tumor formation model. After 10 days, the tumor sizes and body weights of the mice were recorded every 3 days. The tumor volume (V) was calculated using *V* = *π* × *L* × *W*^2^ / 6 (L: Long diameter, W: Wide). The tibial tumor formation model was continuously observed for 3 weeks. At the end of the observation period, all mice were euthanized by chloral hydrate overdose. Then, tibial tumors were dissected and isolated, measured and weighed, and fixed with 4% paraformaldehyde or stored at −80 °C for further analysis.

### Tube formation assay

The OS cell tube formation assay was performed in 96-well plates. Matrigel (BD Biosciences, USA) was fully dissolved at 4 °C, and 50 μL was injected per well and incubated at 37 °C for 30 min. The cells (2 × 10^4^ cell/well) were re-suspended with medium, seeded in each well, and incubated with PBS, recombinant interleukin-6 (IL-6, 25 ng/ml; Peprotech, USA) or stattic (10 μM; Selleck, USA) at 37 °C in 5% CO_2_. Tube-like structures were observed and photographed under an inverted microscope. Tube length was calculated using ImageJ software.

### In vivo Matrigel plug assay

Nude mice (4 weeks old, *n* = 5) were used for in vivo Matrigel plug angiogenesis assays. Each mouse was subcutaneously injected with OS cells (8 × 10^6^ cells) mixed with Matrigel (0.3 mL) into the bilateral ventral region (the left side for the NC group, the right side for the PARK2 group). On day 7, the mice were killed, and the Matrigel plugs were excised and measured for the extent of neovascularization through histological sections and hemoglobin assay.

### Hemoglobin assay

Drabkin’s reagent (Sigma, USA) was used for hemoglobin quantification analysis following the manufacturer’s protocols. In brief, the Matrigel plugs (100 mg) were added to 100 µL RIPA lysis buffer (Sigma, USA), incubated for 30 min, and centrifuged at 4 °C. In 96-well plates, 20 μL of supernatant and 100 μL of Darkin’s solution were added into each well and plates were subsequently incubated for 30 min at room temperature. Then, the plate was detected by a microplate reader at 540 nm absorbance and compared with the blank as reference.

### Statistical analysis

The results were presented as means ± s.e. Student’s *t* tests were performed using GraphPad Prism (v5.0). Data from groups were analyzed by one-way ANOVA. Results were considered statistically significant at *P* < 0.05. Statistical significance was defined as *P < 0.05, **P < 0.01, and ***P < 0.001.
